# The Causes of Hysteria

**Published:** 1864-04

**Authors:** 

**Affiliations:** Prague.; Prague.


### CHRONICLE OF MEDICAL SCIENCE.

*The Causes of Hysteria.* By Professor BROWN-SEQUARD, and Professor JACKSH of Prague.

From notes of a course of lectures recently delivered by this eminent physiologist before the London Medical Society, on nervous diseases, we extract the following suggestive remarks on the causes of hysteria:

"Concerning the essential nature of hysteria, as well as of trembling palsy, Dr. Brown-Sequard confessed that he knows nothing. Some symptoms, however, may be explained—the absence of the sense of touch, for instance: if a cupping glass be applied to a part of the insensible skin, so as to redden it by drawing blood into it, the anæsthesia immediately ceases; in like manner, if an attempt to draw blood by cupping or by leeches be successful, there will be no longer anæsthesia of the par's acted upon. These facts prove that the anæsthesia is not due to a centric cause, but wholly to the want of circulation at the periphery. Referring to the morbid volition, Dr. Brown-Sequard mentioned a case of a young lady (one of his private patients) whose right arm had been completely paralyzed for six years. She had evinced other symptoms of hysteria, and her mother was also liable to this malady. He requested his patient to hold her arms to her sides, and, while doing so, to bend the body forwards. The body having been thus inclined, the right arm remained close to it. Of course, had the paralysis been due to a structural cause, the arm, instead of being retained in its position, would have fallen forwards. Another similar case was that of a girl having a violently shaking hand. Dr. Brown-Sequard stood before her, and said: "I am sure I shall soon be struck by her;" and very soon a movement beyond the ordinary shake occurred, by which he was struck, thus proving the power of the will over the limb. He also described a very singular case of persistent shaking, affecting each part of the body in succession: if, for instance, the arm actually shaking were forcibly held still, the other arm would immediately begin to shake; if the two arms were held, a leg would carry on the movement; if that also were secured, the other would begin; that being also held, the head was violently agitated. If the four limbs and head were all restrained, then the body itself was powerfully shaken. Another curious manifestation of hysteria was spoken of, consisting of swinging the arm round four or five times, then striking the chest with it, the two motions being continued alternately. The affection began immediately after the subject of it had received bad news, and ended as suddenly. The lecturer insisted strongly, that hysteria is a very real, and often a very serious disease. He expressed the opinion that, out of ten hysterical cases, eight would never completely recover, and that even the remaining two would, on some rare occasions, exhibit remnants of the malady. Sometimes muscular atrophy, together with complete and permanent stiffness of joints, follows, and results from hysterical attacks. Calling the attention of his audience to a case of hysterical hypochondriasis, with slight aphonia, the lecturer observed that the malady in this instance was due to poverty of the blood, which, as Dr. Todd has already insisted, is a very frequent cause of hysteria. The appetite of hysterical patients, occupying a good social position, often fails, and thus induces the malady where the tendency to it exists. The more we know of nervous complaints, the more convinced we become of their intimate relation to each other. When transmitted from one generation to another, it frequently happens that they are not transmitted directly, but, as Morell and Pritchard have asserted, a parent having one nervous disease will transmit another to his child, while the several children of one parent will often severally exhibit a distinct form of nervous disease. Not unfrequently one person will present a blending of several forms, as evidenced by a girl whom the lecturer introduced to his audience. When eleven years of age she had scarlet fever, resulting in albuminuria and dropsy. There was extensive ulceration of the neck, and this was followed by a deviation of the spine. There is now a forward and a slight lateral curvature. She was subsequently attacked with what was called rheumatic gout, and at present has great pain in the right arm, but no swelling. In July, 1861, she had hysterical fits, which reappeared some months afterwards, and finally emerged into genuine epilepsy. She has also a slight choreic movement of the arm and hand. The movement is not tremulous, and the will seems to have some share in it. The eyes protrude a little; both pupils are large; and, owing to an irritation of the sympathetic nerve, one is larger than the other. Another case exhibiting a like blending of nervous diseases, was also shown: a man, aged 33, who had convulsions when three or four months old, preceded by hemorrhage from the bowels, afterwards became paralyzed on the left side, where the convulsive movements had been most powerful. Epilepsy supervened, and was followed by jerkings and twitchings of the limbs, and a peculiar contorting spasmodic movement, which still continues, of the arm and hand. The arm has been more affected from the first than the leg, and is considerably shorter than that of the other side. The hand and fingers may be extended if the force be applied gently; but on its withdrawal the original position is immediately resumed. The treatment in this case simply consists in the application of circular blisters to the arm, in the hope of producing a modification of nutrition in the nervous centre. This lecture was closed by some remarks upon infantile paralysis; but as they were resumed in the following one, we reserve them until our next report.

That the pathology of hysteria, as suggested by Brown-Sequard, may be attributable to disturbances of the functions of the peripheral nerves, we append an extract from a paper on this subject, read before the Congress of German naturalists and physicians, at Carlsbad, December, 1862, by Professor Jacksh, of Prague, in which he adverted principally to anodynia and cutaneous anæthesia. In the former affection the nerves had lost the power of transferring the perception of pain to the encephalon, so that the prick of a pin blisters, and Faradisation, by means of wire-brushes, remained unperceived; while in the latter the sense of temperature, pressure, and locality were wanting. There were different degrees of both affections; the anæsthesia was either complete or incomplete. With regard to its extent, there was the greatest variety. Anodynia frequently occupied only one half of the body, but it sometimes extended beyond the median line; in other cases it was confined to a very small circumference. Anodynia of the tongue was often associated with that of the skin. The sensibility of the muscles was only seldom increased, but generally diminished or entirely gone. This affection might be likewise either partial or general, and did not follow certain courses of nerves; as, for instance, the trapezius might be affected, while the sterno-cleido-mastoid remained in its normal condition. The electro-muscular contractility and the voluntary movements were, generally, not impaired; sometimes, however, he had seen them deficient, especially in the lower extremities. The mucous membranes might be affected in a similar manner; there might be loss of smell and taste, anodynia of the Schneiderian membrane, of the mucuous membrane of the aerial passages, the vagina, and the rectum, anæsthesia of the retina, etc. The duration of the affection varied from several days to several years. It generally ensued after emotion, and sometimes disappeared after cold aspersions. Amongst the diseases most frequently associated with anodynia, Professor Jacksh had observed chorea, epilepsy, catalepsy, somnambulism, melancholia, paralysis, and contractions, fits of laughing, vomiting, neuralgia, disphagia, Brodie's tumor of the subcutaneous cellular tissue, and paroxysms of apparent ague. The affection was more frequent in females than in males, more in Jews than in Christians, and was chiefly observed in persons between twelve and thirty years of age. The prognosis was not very favorable, as relapses were frequent. The medicines chiefly to be employed were zinc, quinine, secale cornuta, morphium. Cold aspersions, the douche, etc., were also serviceable; but by far the best remedy was Faradisation, especially if paralysis, contractions, and neuralgia were at the same time present.—*Medical Times and Gazette.*

